# Pregnancies complicated with functioning adrenal adenomas causing severe obstetric outcomes: a 20-year experience at a tertiary center

**DOI:** 10.1186/s12902-024-01655-9

**Published:** 2024-07-24

**Authors:** Caixia Zhu, Shiqin Cai, Xue Zhong, Linhuan Huang

**Affiliations:** Department of Obstetrics and Gynecology, The First Affiliated Hospital of Sun Yat-sen University, Zhongshan er Road, No.58, Guangzhou, 510080 China

**Keywords:** Adrenal adenoma, Pregnancy, Obstetric, Neonatal, Outcome

## Abstract

**Background:**

Functioning adrenal adenoma during pregnancy is rare, and the diagnosis is challenging owing to unspecific symptoms and restricted investigations. The obstetric outcomes of patients who undergo surgery during pregnancy or who receive only medical treatment are poorly described.

**Objective:**

The aim was to investigate the associations between functioning adrenal adenomas and obstetric outcomes.

**Methods:**

A retrospective study was performed in a tertiary center over 20 years. The clinical characteristics, management and obstetric outcomes of the diagnosed pregnant women were reviewed.

**Results:**

A total of 12 women were diagnosed with functioning adrenal adenomas during pregnancy from January 2002 to September 2022. Eight women had cortisol-secreting adrenal adenomas, two had excessive catecholamine secretion, and two had primary aldosteronism. The initial symptoms of adrenal adenoma during pregnancy included hypertension or preeclampsia, gestational diabetes mellitus or prepregnancy diabetes mellitus, hypokalemia and ecchymosis. Four women underwent adrenalectomy during pregnancy, while 8 women received only medical therapy. Preterm birth occurred in all patients who received medicine, whereas 1 patient who underwent surgery experienced preterm birth. Among the 8 women in the medical treatment group, 3 had neonates who died.

**Conclusions:**

Once hypertension, hyperglycemia and hypokalemia occur during the 1st or 2nd trimester, pregnant women with adrenal adenomas should be evaluated via laboratory and imaging examinations. The maternal and fetal outcomes were unpredictable owing to the severity of adrenal adenoma, particularly in patients who received only medical treatment. Adrenalectomy should be recommended during pregnancy.

**Supplementary Information:**

The online version contains supplementary material available at 10.1186/s12902-024-01655-9.

## Introduction

Functioning adrenal adenoma, which often causes infertility owing to ovulatory dysfunction, is rare during pregnancy.Functioning adrenal adenoma increased the risk of adverse maternal and fetal outcomes, including preeclampsia, hypertensive crisis, hyperglycemia, heart failure and other severe complications [1–3]. It is challenging to diagnose and treat patients with adrenal adenomas without a consensus on strategy. First, the absence of a typical clinical presentation and leading signs are the main causes of the difficulty of diagnosis. Second, experimental tests are lacking for pregnant women because hormone assays are rarely performed during pregnancy [4]. In addition, imaging investigations are limited to pregnant women to avoid fetal radiation exposure. The rarity of functioning adrenal adenoma during pregnancy has contributed to a delay in diagnosis. Typically, adrenalectomy is required for patients with adrenal adenomas [5]. However, reports of adrenalectomy during pregnancy are rare, leading to the challenge of managing pregnancies complicated with adrenal adenomas.

The purpose of the present study was to report the experience of a tertiary hospital experience with pregnancies complicated by functioning adrenal adenomas and explore their management and short- and long-term obstetric outcomes.

## Materials and methods

A retrospective study was conducted in a tertiary hospital from January 2002 to September 2022. A total of 12 patients who were diagnosed with functioning adrenal adenomas during pregnancy were enrolled. Data on the patients’ clinical characteristics, management and obstetric outcomes were obtained. Laparoscopic adrenalectomy was performed for all patients during pregnancy or the postpartum period. The diagnosis of functioning adrenal adenoma was confirmed by a pathologist. The study was approved by the Ethics Committee of the First Affiliated Hospital, Sun Yat-sen University. Written informed consent for the publication of their clinical details was obtained from the patients. We certify that the study was performed in accordance with the Declaration of Helsinki and its later amendments. Clinical, hormonal and radiological evaluations with ultrasonography, computed tomography (CT) and magnetic resonance imaging (MRI) were performed for all patients. During the early period of this study, a CT scan was recommended for pregnant women for whom an adrenal tumor was highly suspected upon clinical presentation and if there was no positive finding on ultrasound. A multidisciplinary team (MDT), including obstetricians, endocrinologists, surgeons, pediatricians, radiologists and anesthesiologists, discussed the management of functioning adrenal adenomas. All patients received symptomatic medications such as antihypertensive and diabetic medications, and patients who underwent surgery were treated with pancreatic enzyme supplementation.

## Results

The demographics and clinical characteristics of the patients are summarized in Table 1. A total of 12 patients were diagnosed with functioning adrenal adenomas during pregnancy over a 20-year inclusion period. Briefly, the median age of the patients at the time of diagnosis was 30 years (23–37 years). Eleven patients conceived naturally, and 1 patients assisted in reproduction. Among the 12 included patients, 4 patients were multiparous, and 8 patients were nulliparous. The initial symptoms of the above mentioned pregnant women included hypertension or preeclampsia (*n* = 9), gestational diabetes mellitus or prepregnancy diabetes mellitus (*n* = 7), hypokalemia (*n* = 11) and ecchymosis (*n* = 7). Hypertension or diabetes mellitus occurred in 3 patients before pregnancy. Chronic hypertension was detected in 3 patients, while hypertension was detected between 20 weeks and 33 weeks of gestation. A total of 11 patients tolerated hypokalemia as early as 7^+ 3^ weeks of gestation.

**Table 1:** The demographicesarean and clinical characteristics of 12 pregnancies with functioning adrenal adenoma

Pts #	Age (years)	Conception	Parity	The gestational age at onset of symptoms (wks)	Symptoms	The gestaional age at diagnosis (wks)	Diagnosis
1	26	nature	nulliparous	33	central obesity, moon-shaped face, purple striae, hypertension, hypokalemia	34	Cushing’s syndrome
2	27	nature	nulliparous	24 + 3	central obesity, moon-shaped face, purple striae, hypertension, hypokalemia, hyperglycemia	31 + 2	Cushing’s syndrome
3	29	nature	multiparous	26 + 4	central obesity, moon-shaped face, purple striae, hypertension, hypokalemia, hyperglycemia	27	Cushing’s syndrome
4	27	nature	multiparous	18	central obesity, moon-shaped face, purple striae, hypertension, hypokalemia, hyperglycemia	20 + 3	Cushing’s syndrome
5	34	nature	nulliparous	13	central obesity, moon-shaped face, purple striae, hypokalemia, hyperglycemia	19	Cushing’s syndrome
6	33	nature	nulliparous	7 + 3	central obesity, moon-shaped face, purple striae, hypertension, hypokalemia, hyperglycemia	18 + 1	Cushing’s syndrome
7	31	IVF-ET	nulliparous	26	central obesity, moon-shaped face, hypertension, hypokalemia, hyperglycemia	29 + 4	Cushing’s syndrome
8	32	IVF-ET	multiparous	11	central obesity, moon-shaped face, purple striae, hypertension, hypokalemia, hyperglycemia	13 + 4	Cushing’s syndrome
9	36	nature	multiparous	pre-pregnancy	hypertension	29 + 4	primary aldosteronism
10	23	nature	nulliparous	13 + 6	hypertension, hypokalemia	13 + 6	pheochromocytoma
11	37	nature	nulliparous	22 + 3	hypertension, hypokalemia	29 + 6	primary aldosteronism
12	27	nature	nulliparous	20	hypertension, hypokalemia	28 + 6	pheochromocytoma

As shown in Table 2among the 12 patients, Patients 1 to 8 were diagnosed with Cushing’s syndrome. All of the above 8 patients had central obesity and moon-shaped faces. The remaining clinical findings included hypertension, purple striae and hypokalemia. In addition, Patients 9 and 11 were diagnosed with primary hyperaldosteronism, which was complicated only with hypertension. Patients 10 and 12 were diagnosed with pheochromocytoma, which involved typical symptoms such as hypertension and hypokalemia. Two patients were diagnosed during the first trimester, 4 during the second trimester and 6 during the third trimester.

**Table 2:** The laboratory and imageological examination of 12 pregnancies with functioning adrenal adenoma

Pts #	Cortisol (0am) µg/dL	Cortisol (8 am) µg/dL	Urinary free cortisol (µg/24 h)	ACTH (pmol/L)	Serum potassim (mmol/L)	Aldosterone (pg/mL)	Ultrasound*	MR*	CT*
1	86.2	73.5	1575.0	0.22	2.70	50.2	left side	-	-
2	40.3	37.9	782.0	1.01	3.14	47.5	right side	-	right side
3	28.8	30.8	627.3	1.53	2.50	78.6	left side	-	left side
4	25.66	26.6	978.4	2.4	2.86	32.5	-	right side	-
5	39.46	38.59	2639.9	0.22	3.14	45.6	left side	--	
6	30.6	30.1	1094.1	0.33	2.28	67.8	-	right side	-
7	30.0	29.9	3542.8	0.12	2.39	27.0	left side	left side	-
8	22.2	24.3	504.0	0.27	3.56	111.0	left side	left side	-
9	88.2	50.4	445.6	2.2	2.60	815.1	left side	-	left side
10	12.3	23.2	528.5	1.2	4.05	321.5	right side	-	-
11	9.2	12.8	421.2	13.69	2.42	1876.2	-	right side	-
12	9.5	23.1	342.1	0.27	3.96	230.2	right side	right side	-

Reference range:

Cortisol (0am): ; 2.90–19.40 µg/dL; Cortisol (8am): 2.90–19.40 µg/dL; Urinary free cortisol (24 h): 4.30–176.00 µg/24 h; ACTH: 1.60–13.90 mmol/L; Aldosterone: 10.00−160.00pg/mL

* Ultrasound,** MR or CT scan showed that adrenal adenoma on the left or right side**

In present study, four patients underwent laparoscopic adrenalectomy during pregnancy (Patients 4, 5, 6, 10), whereas the remaining eight patients underwent laparoscopic adrenalectomy in the postpartum period. According to the pathological data, eight of the 12 adrenal adenoma patients had cortisol-secreting adenomas (Patient 1–8), whereas two had excessive catecholamine secretion (Patient 9, 11) and two had primary aldosteronism (Patient 10, 12). No micro/macronodular adrenal hyperplasia was detected in the patients with Cushing’s syndrome. Furthermore, there were no cases due to bilateral aderenal adenoma.

Table 3 shows the maternal outcomes. Labetalol was used to control hypertension. Potassium supplement was used to control hypokalemia. Further, ursodeoxycholic acid has been recommended for the patients with intrahepatic cholestasis of pregnancy. Among the 4 patients who underwent laparoscopic adrenalectomy during pregnancy, two patients experienced stillbirth (Patient 5 and Patient 10). Two patients underwent cesarean section at 38 weeks of gestation and at 36 weeks of gestation. Among the 8 remaining patients who underwent laparoscopic adrenalectomy a few months after delivery, only one patient delivered vaginally, and the remaining 7 patients underwent cesarean section. Overall, all 8 patients gave birth before 37 weeks of gestation, including 4 who delivered late-preterm (34–36 + 6 weeks) infants and 4 who delivered early-preterm (28–33 + 6 weeks) infants.

**Table 3:** The maternal outcomes of 12 pregnancies with functioning adrenal adenoma

Pts #	Gestational age at delivery (wks)	Obstetric outcome	Delivery mode	Hypertension /preeclampsia	GDM/PGDM*	ICP#	Thrombocytopenia	PROM**	Hypokalemia	Medical Treatment
1	34	late preterm	cesarean	yes	no	no	no	no	yes	labetalol, potassium supplement
2	31 + 3	preterm	vaginal	yes	yes	no	no	yes	yes	labetalol, potassium supplement
3	31	preterm	cesarean	yes	yes	no	no	no	yes	labetalol, potassium supplement
4	38	term	cesarean	no	yes	no	no	no	yes	potassium supplement
5	22 + 2	abortion	-	no	yes	no	no	yes	yes	potassium supplement
6	36	late preterm	cesarean	yes	yes	no	no	no	yes	labetalol, potassium supplement
7	32 + 4	preterm	cesarean	no	yes	yes	yes	no	yes	ursodeoxycholic acid, potassium supplement
8	36 + 5	late preterm	cesarean	yes	yes	yes	yes	no	yes	labetalol, ursodeoxycholic acid, potassium supplement
9	29 + 4	preterm	cesarean	yes	no	no	no	no	no	labetalol
10	19+	abortion	-	yes	no	no	yes	yes	yes	labetalol, potassium supplement
11	34 + 6	late preterm	cesarean	yes	no	no	no	no	yes	labetalol, potassium supplement
12	35 + 3	late preterm	cesarean	yes	no	no	no	no	yes	labetalol, potassium supplement

*GDM/PGDM: getational diabetes mellitus/ pre-existing gestational diabetes mellitus

#ICP: intrahepatic cholestasis of pregnency

**PROM: prelabor rupture of membranes

With respect to neonatal outcomes (Table 4), 9 patients had live-born infants, and two patients experienced fetal loss after surgery. There was no difference in neonatal sex among all the newborns. All patients in the medical treatment group had preterm infants who were admitted to the NICU. In the medical treatment group, more patients delivered neonates with low birth weights. Neonatal asphyxia occurred in only two neonates born to patients in the medical treatment group. During long-term follow-up, Patients 2, 3 and 9 experience neonatal death owing to preterm birth. Patients 3 and 7 delivered neonates who had malformations. The child of Patient 4 developed nephrotic syndrome at 2 years of age.

**Table 4:** The neonatal outcomes of 12 pregnancies with functioning adrenal adenoma

Pts #	Time at surgery (wks)	Pathology	Gestational age at delivery (wks)	Neonatal birth weight (g)	Gender*	Low birth weight	Neonatal asphyxia	NICU admission	Perinatal outcome	Others
1	postpartum	cortisol-secreting adenoma	34	2600	M	no	yes	yes	yes	
2	postpartum	cortisol-secreting adenoma	31 + 3	2700	F	no	yes	yes	neonatal death	malformation
3	postpartum	cortisol-secreting adenoma	31	1100	M	yes	no	yes	neonatal death	
4	20+	cortisol-secreting adenoma	38	2750	M	no	no	no	alive	nephrotic syndrome
5	21+	cortisol-secreting adenoma	22 + 2	-	-	-	-	-	-	-
6	19+	cortisol-secreting adenoma	36	2000	F	yes	no	yes	alive	
7	postpartum	cortisol-secreting adenoma	32 + 4	2170	M	yes	no	yes	alive	duplex kidney
8	postpartum	cortisol-secreting adenoma	36 + 5	2500	F	no	no	yes	alive	
9	postpartum	primary hyperaldosteronism	29 + 4	980	F	yes	-	no	neonatal death	
10	17 + 3	pheochromocytoma	19+	-	-	-	-	-	-	-
11	postpartum	primary hyperaldosteronism	34 + 6	2250	M	yes	no	yes	alive	
12	postpartum	pheochromocytoma	35 + 3	2820	M	no	yes	yes	alive	lateral ventricle hemorrhage

*M: male; F:female

## Discussion

In the present series of 12 patients with functioning adrenal adenomas diagnosed during pregnancy, we reported that the management of functioning adrenal adenoma was still challenging despite performing surgery during pregnancy. We found that hypertension, hyperglycemia and hypokalemia might be considered the symptom triad of functioning adrenal adenomas, especially in the second trimester of pregnancy. Patients who received medical treatment, including more than 75% of patients with preeclampsia, preterm birth and other severe complications, had an increased risk of severe obstetric outcomes. The rates of low neonatal birth weight and NICU admission were increased in patients who received medical treatment, especially those with Cushing’s syndrome or pheochromocytoma.

In our study, most cases of functioning adrenal adenomas were cortisol-secreting adrenal adenomas. The clinical symptoms and signs of Cushing’s syndrome during pregnancy are atypical owing to the anatomical changes that occur during pregnancy, leading to delayed diagnosis during pregnancy. Clinical evaluations have also been performed during pregnancy, identifying symptoms such as hypertension, diabetes mellitus and obesity owing to adrenal adenomas [6]. In our study, most patients with Cushing’s syndrome presented with central obesity, moon-shaped faces, hypertension, and hypokalemia, whereas hypertension, gestational diabetes mellitus and hypokalemia were detected in patients with pheochromocytoma or primary hyperaldosteronism. Pheochromocytoma secretes catecholamine hormones that inhibit sodium reabsorption in the renal tubules, causing large amounts of sodium and water to be excreted from the body. As more sodium is excreted in the urine, the amount of sodium in the body decreases, leading to hyponatremia. In turn, hyponatremia also leads to increased excretion of potassium ions, which leads to hypokalemia.

Therefore, the diagnosis of functioning adrenal adenoma is challenging. Unlike prepregnancy diagnoses, the diagnostic strategy during pregnancy was based on physiological hypercortisolism. The loss of rhythmicity of cortisol secretion was detected in women with pregnancies complicated by Cushing’s syndrome [7]. In addition, urinary cortisol or catecholamine levels are increased in women with adrenal adenomas during pregnancy, which is helpful for making a diagnosis[8]. If ultrasound does not detect any findings, MRI should be performed for patients suspected of having adrenal lesions.

Haitham [8] reported that the risk of preeclampsia was greater in patients with Cushing’s syndrome than in controls. Similarly, we also found that 9 patients (75%) had preeclampsia or severe hypertension in our study; these patients were at increased risk. In addition, only one patient delivered at term in our study. In contrast, the authors reported that the risks of preterm delivery and gestational diabetes mellitus were similar in patients with Cushing’s syndrome and in the control group. In line with the findings of the present study, Caimari [9] reported a high risk of hyperglycemia in pregnant patients with Cushing’s syndrome. In our study, one patient developed acute pancreatitis and was in a life-threatening situation. Previously, only one case report [10] reported acute pancreatitis in a nonpregnant women with Cushing’s syndrome. It is difficult to illustrate a potential association between adrenal adenoma and acute pancreatitis.

Owing to the limited experience and rarity of cases during pregnancy, the management of functioning adrenal adenomas during pregnancy is still limited. In our study, only 4 patients underwent laparoscopic adrenalectomy during pregnancy. The decision to perform surgery was made by the MDT and the patients’ will. In the present study, two patients who underwent laparoscopic adrenalectomy during pregnancy delivered between 36 weeks and 38 weeks of gestation, while two patients (50%) experienced miscarriage. In the literature, 10 patients with Cushing’s syndrome due to adrenal adenoma underwent laparoscopic adrenalectomy during pregnancy, and 3 patients (30%) delivered after 37 weeks of gestation [11–20]. Among these 10 patients, only one experienced miscarriage. Sébastien and colleagues reviewed the cases of 12 patients who underwent laparoscopic adrenalectomy during pregnancy before 20 weeks of gestation, 10 patients had live-born infants, and 6 (50%) patients had term deliveries. Laparoscopic adrenalectomy should be performed before 24 weeks of gestation. Open adrenalectomy should not be performed after 24 weeks of gestation due to the enlarged uterus. In the guidelines of the *Society of American Gastrointestinal and Endoscopic Surgeons* (SAGES), laparoscopic adrenalectomy is recommended during any trimester of pregnancy [21]. However, adrenalectomy performed during the third trimester of pregnancy is still controversial, and no data are available. It is difficult to postpone adrenalectomy after delivery because of severe complications.

Medical management was provided for the remaining 8 patients. In all cases, vitamin D supplementation, calcium tablets and/or antihypertensive therapy were used during pregnancy. Phenoxybenzamine was used for patients with pheochromocytoma to control the hypertension before operation. All these patients received pancreatic enzyme supplementation before laparoscopic adrenalectomy, regulating the glucose and lipid metabolism. Labetalol were used to control blood pressure for Patient 1, 2, 3, 6, 8, 9, 10, 11 and 12, but were inefficient. For preeclampsia caused by adrenal adenomas, various medicines, including nifedipine, nitroglycerin and magnesium sulfate, have been used during pregnancy, especially before 34 weeks of gestation. Medical treatments for conditions such as severe preeclampsia, thrombocytopenia and acute pancreatitis carry a potential risk for adverse maternal and fetal outcomes [22]. All patients who received medical therapy had preterm delivery. An unpredictable clinical course might be a potential factor for preterm birth. Typically, once symptoms causing severe complications occur, termination of pregnancy should be performed. Preterm birth, particularly before 32 weeks of gestation, is associated with neonatal morbidity and morbidity. It is still challenging to choose an optimal scheme without guidelines for choosing between surgery and medical therapy.

In our experience, adverse neonatal outcomes, including low birth weight, neonatal asphyxia and NICU admission, occur in patients with adrenal adenomas. Two miscarriages (50%) occurred in the group who underwent adrenalectomy during pregnancy, whereas 2 patients experienced miscarriage in the group who received medical therapy. Three newborns in the group that received only medical therapy died. One newborn had fetal malformations, and the remaining newborns experienced severe complications due to premature delivery. Poor neonatal outcomes seem to be associated with premature birth. Younes [23] reported that successful treatment of hypercortisolism did not improve fetal outcomes. In accordance with a previous report, adrenalectomy during pregnancy seemed to improve neonatal outcomes. During follow-up, The child of Patient 4 developed nephrotic syndrome at 2 years of age, and the child of Patient 7 had a duplex kidney. It is difficult to say whether the risk of long-term complications in newborns is increased in pregnancies complicated by adrenal adenomas.

In our study, new-onset hypertension, hyperglycemia and hypokalemia during the second trimester might indicate a lesion located in the adrenal gland. Unlike hyperemesis gravidarum, most cases are complicated with hypokalemia in the 2nd trimester. Similarly, hypertension and hyperglycemia also occur in the 1st or 2nd trimester. Adrenal adenomas can secrete cortisol, which affects blood sugar metabolism and balance, resulting in hyperglycemia and hypertension earlier during pregnancy [24]. In addition, functioning adrenal adenomas, which secrete aldosterone or catecholamine, can cause hypertension and hypokalemia as early as the onset of adrenal adenomas [25]. Furthermore, once patients have central obesity and moon-shaped faces, hypertension, hyperglycemia and hypokalemia can occur, suggesting Cushing’s syndrome. If patients have hypertension, gestational diabetes mellitus or hypokalemia, they may be considered to have pheochromocytoma or primary hyperaldosteronism [26, 27]. Therefore, ultrasound or MRI of the adrenal gland is recommended for pregnant women with hypertension, hyperglycemia and hypokalemia.

In the present study, most patients chose medical treatment, and all of these patients experienced preterm birth. The causes of pregnancy termination were severe complications such as preeclampsia, intrahepatic cholestasis of pregnancy (ICP), thrombocytopenia and cholecystitis. Compared with that in the surgery group, the maternal morbidity rate in the group that received only medical treatment was increased. However, these findings should be interpreted with caution owing to the small number of included patients. Considering the uncertain risk of severe complications of adrenal adenoma and adverse neonatal outcomes, we suggest that patients undergo laparoscopic adrenalectomy during pregnancy [28–30]. Based on the present experience, laparoscopic adrenalectomy should not be delayed unnecessarily owing to adverse maternal and neonatal outcomes.

In view of the rarity of adrenal adenomas during pregnancy, there are several limitations to the present study. First, the retrospective experience is limited by the small sample size (*n* = 12). In addition, the decision of surgery bias over the study period could not be excluded. All these limitations precluded us from drawing evidence-based conclusions for the management of adrenal adenomas during pregnancy. Nevertheless, the present study represents a large cohort of pregnant women with adrenal adenomas, suggesting that surgical management is safe. It is difficult to make a decision regarding the appropriate timing of adrenalectomy for adrenal adenoma during pregnancy, which requires a multidisciplinary approach and the desire of patients.

When required by the maternal and fetal situation, adrenalectomy during pregnancy is associated with an excellent outcome for mothers but some expected morbidity for neonates. The management of pregnant patients with these lesions depends on the timing of delivery and the severity of the underlying adrenal disease. Adrenalectomy should be performed ideally during the first and second trimesters and should be performed laparoscopically if possible after a MDT. Maternal outcomes are acceptable, and fetal outcomes are affected by the underlying disease, with the worst outcomes in patients for whom adrenalectomy is indicated for malignant lesions or Cushing’s syndrome. Management of these more difficult patients should initiate referral to a specialized referral multidisciplinary center where appropriate specialty surgical, obstetric, and pediatric care is available.

## Conclusions

In summary, functioning adrenal adenomas during pregnancy are extremely rare, and the diagnosis is challenging. Owing to the overlapping clinical features of both healthy pregnant patients and those whose pregnancies are complicated with adrenal adenomas, it is necessary to determine an accurate index of suspicion. Imaging and laboratory examinations should be performed when hypertension, hyperglycemia and hypokalemia simultaneously occur during pregnancy. The possible adverse maternal and fetal outcomes are broad, and the patients have high risk, especially pregnant patients who only receive only medical treatment. Hence, individual management should be carried out by an MDT.

### Electronic supplementary material

Below is the link to the electronic supplementary material.

Supplementary Material 1

## Data Availability

The datasets generated and/or analysed during the current study are not publicly available due patient privacy but are available from the corresponding author on reasonable request.
