# Supplementary material for: Pregnancies complicated with functioning adrenal adenomas causing severe obstetric outcomes: a 20-year experience at a tertiary center
Source: BMC Endocr Disord. 2024 Jul 24;24:122. doi: 10.1186/s12902-024-01655-9 (PMC11267956; doi:10.1186/s12902-024-01655-9)
Supplement: Supplementary file 1 — Supplementary Material 1 [file 12902_2024_1655_MOESM1_ESM.docx]

**Supplemental Table 1**

| **Literature** | **Previous findings** | **Present findings** |
| --- | --- | --- |
| Andreescu C E (2017) | The clinical evaluation such as hypertension, diabetes mellitus and obesity owing to adrenal adenoma was also observed during pregnancy. | In present study, most of cases with Cushing’s syndrome suffered from central obesity, moon-shaped face, hypertension, and hypokalemia, while hypertension, gestational diabetes mellitus and hypokalemia were detected in cases with pheochromocytoma or primary hyperaldosteronism. |
| Hamblin Ross (2022) | The loss of rhythmicity of cortisol-secretion was detected in pregnancy complicated with Cushing’s syndrome | The same results |
| Baghlaf Haitham A (2022) | The urinary cortisol or catecholamines increased in the adrenal adenoma during pregnancy, which help us to make a diagnosis. | The same results |
| Caimari Francisca (2017) | Caimar evaluated high risk of hyperglycemia in pregnancies with Cushing’s symdrome. | In present study, 7 cases suffered from hyperglycemia in pregnancies with Cushing’s symdrome. |
| Bouali M (2021) | Only one case reported acute pancreatitis complicated in non-pregnant women with Cushing’s syndrome. | In present study, one cases suffered from pancreatitis during pregnancy. |
| Chang Ikjin (2013)  Blanco C (2006)  Zhang Xin (2019)  MacGibbon A L (1995)  Wang Wei (2015)  Lo K W (1998)  Martínez García R (2016)  Lu Zhenquan (2019)  Trinh Anne (2016)  Kim Hwi Gon (2003) | In view of the existing literature that reported 10 patients with Cushing’s syndrome due to adrenal adenoma, who took laparoscopic adrenalectomy during pregnancy, 3 cases (30%) delivered after 37 weeks of gestation | In present study, 8 cases suffered from Cushing’s syndrome and only one case delivered after 37 weeks of gestation. |
| Pearl Jonathan P (2017) | In the guildline of *Society of American Gastrointestinal and Endoscopic Surgeons* (SAGES), laparoscopic adrenalectomy is recommend during any trimester of pregnancy | In present study, adrenalectomy was performed during the 2^nd^ trimester. |
| Eschler Deirdre Cocks (2015) | The medical treatments carried a potential risk for adverse maternal and fetal outcomes, such as severe preeclampsia, thrombocytopenia and acute pancreatitis | All patients received medicine therapy had preterm delivery. |
| Brue Thierry (2018) | Younes identified that successful treatment of hypercortisolism did not improve the fetal outcomes. | In the follow-up, we found one case suffered from nephrotic syndrome at 2 years old and one case had duplex kidney. It’s hard to say the risk of long-term comolications of newborn increased in the pregnancies with adrenal adenoma. |
| Younes Nada (2022)  Parksook Wasita Warachit (2022)  [Tomáš Zelinka](https://pubmed.ncbi.nlm.nih.gov/?term=Zelinka+T&cauthor_id=32114578) (2020)  Lotgering F K (1986) | Adrenal adenoma would secrect cortisol which affects the blood sugar metabolism and balance, resulting hyperglycemia and hypertension earlier than pregnancy complicated with hypertension or gestational diabetes mellitus. Besides, adrenal adenoma which secrects aldosterone or catecholamine, leads to hypertension and hypokalemia as early as onset of adrenal adenoma | If patiens suffered from hypertension, gestational diabetes mellitus and hypokalemia, it might be considered as pheochromocytoma or primary hyperaldosteronism |
| Delić Ratko (2022)  Kyriakos Georgios (2021)  Kazi Stephanie D (2020) | Considering uncertain risk of severe complications of adrenal adenoma and adverse neonatal outcomes, the patients should take laparoscopic adrenalectomy during pregnancy | In the present experience, laparoscopic adrenalectomy should not be delayed unnecessarity owing to the adverse maternal and neonatal outcomes. |
